# All‑cause excess mortality in Germany peaked during the late‑2022 influenza period, exceeding peaks during SARS‑CoV‑2 waves (2020–2023)

**DOI:** 10.1371/journal.pone.0335982

**Published:** 2026-06-17

**Authors:** Ursel Heudorf, Bernd Kowall

**Affiliations:** 1 MDRO-Network Rhine-Main, Dietzenbach, Germany; 2 Institute for Medical Informatics, Biometry and Epidemiology, University Hospital Essen, Essen, Germany; Chinese University of Hong Kong, HONG KONG

## Abstract

**Introduction:**

Numerous studies have examined mortality during the SARS-CoV-2 pandemic in Germany and worldwide. In Germany, excess mortality was highest in 2022 compared to the other pandemic years. In a small-scale analysis in Frankfurt am Main, Germany, the excess mortality 2022 was associated with an influenza wave at the end of the year. The aim of this study was to investigate this for the whole of Germany.

**Methods:**

We used publicly available data for the number of deaths, for the population data and for notifications of SARS-CoV-2 and influenza. Standardized mortality ratios (SMR) were estimated for Germany for the years 2020–2023, for seven SARS-CoV-2 waves and for the influenza wave at the end of 2022. Expected numbers of deaths were estimated by two methods: in the first, average mortality in the pre-pandemic years 2016–2019 was used; in the latter, an exponential extrapolation was used to consider the increase in life expectancy.

**Results:**

Relative excess mortality was highest in 2022 (SMR = 1.069 (95% confidence interval: 1.066–1.071) by method 1, SMR = 1.094 (1.092–1.096) by method 2). During the influenza wave from calendar week 47 / 2022 to calendar week 1 / 2023, the SMR was higher than that of any SARS-CoV-2 wave: SMR = 1.252 (95% CI: 1.246–1.258) by method 1, and SMR = 1.374 (95% CI: 1.367–1.380) by method 2. Among all waves considered, the mean number of excess deaths per week was highest during the influenza wave by both methods (5,043, and 6,812, respectively). Age-stratified analyses showed that the excess mortality during the influenza wave at the end of 2022 was highest in individuals aged 70 years and older.

**Discussion:**

During an influenza wave at the end of 2022, excess mortality was higher than in any SARS-CoV-2 wave in 2020–2022 in Germany. Because this study is based on all‑cause mortality and population‑level surveillance indicators, it cannot establish causation or quantify the proportion of excess deaths directly attributable to influenza infection.

## Introduction

Numerous studies have examined mortality during the SARS-CoV-2 pandemic – in Germany and worldwide [1–4], exhibiting very large differences between different countries. Because of the great uncertainties in the notification of SARS-CoV-2-related deaths and in terms of documenting the causes of death, the calculation of overall mortality is generally preferred to the calculation of mortality from COVID-19 [5,6]. Overall mortality reflects not only the direct impact of COVID-19, but also indirect effects such as the measures taken during the pandemic (non-pharmaceutical and pharmaceutical, i.e., vaccination); other additional influencing factors cannot be ruled out.

Total excess mortality showed large differences depending on the calculation methods, such as the choice of age groups for age standardization, the choice of the pre-pandemic reference period, and the procedure for extrapolating mortality rates from the reference period to the index period [7–9]. For this reason, it was recommended that different methods always be used in order to obtain robust statements on excess mortality.

For Germany, the strong dependence of excess mortality on choice of methods was demonstrated by Levitt et al. who gathered results from six different studies with excess death estimates ranging from 54,740–203,000 [8]. The same authors used 66 different reference periods to estimate excess mortality which led to an average relative excess mortality of 1.1% for Germany for 2020 and 2021 – results ranged from −4.3% to 4.6% [9]. Rockenfeller et al. reported an under-mortality of 18,500 death cases in 2020, a moderate excess mortality of 7,000 death cases in 2021 and a strong excess mortality of 41,200 cases in 2022 [10]. Accordingly, Kuhbandner et al. found a strong increase in the number of excess deaths for the period from April 2022 to March 2023 with 78,493 excess deaths [11].

Looking at the pandemic since the first week of 2020, the cumulative excess mortality rate in Germany was 2.4% by the end of 2021; it rose to 3.8% in 2022 [3].

In a small-scale analysis in Frankfurt am Main, Germany, a considerable and significant excess mortality in 2022 had been detected [12], whereas there was no significant deviation in overall mortality for Frankfurt am Main for the years 2020 and 2021 [13]. This excess mortality in 2022, however, was not associated with SARS-CoV-2 waves, but with an influenza wave in the last 6 weeks of 2022. The excess mortality during that influenza wave in Frankfurt am Main was greater than during all SARS-CoV-2 pandemic waves combined [12].

The aim of this hypothesis-driven study was to investigate whether the result found for Frankfurt am Main could be confirmed for the whole of Germany, and whether the high excess mortality in Germany in 2022 could be associated with an influenza wave.

## Materials and methods

We used publicly available data which are available as supplementary tables (S1 File, S2 File, S3 File). The death cases were obtained from the homepage of the Federal Statistical Office [14]. Mid-year population data on an annual average were retrieved from the online database of Federal Health Reporting [15]. The SARS-CoV-2 and influenza reporting data per week were retrieved using a SurvStat query [16].

The definitions of the different SARS-CoV-2 waves in Germany were obtained from Tolksdorf et al. [17]: Wave 1 calendar week (CW) 10–20/2020; Wave 2 CW 40/2020–8/2021; Wave 3 CW 9–23/2021; Wave 4 CW 31–51/2021, Wave 5 CW 52/2021-21/2022, wave 6 starting in CW 22 [17]. Because further definitions are not yet published by the Robert Koch-Institute, we defined the duration of Waves 6 and 7 according to the SARS-CoV-2 notification rates: Wave 6 CW 22–33/2022 and wave 7 CW 34–46/2022. In addition, we used influenza notifications from SurvStat query to derive the interval for an influenza wave from CW 47/2022–01/2023 [16]. In a sensitivity analysis, an alternative time interval for the influenza wave at the end of 2022 was used which was suggested by Norgaard et al. (CW 48 / 2022–2 / 2023) [18].

Analyses were performed for the whole years of 2020–2023, for the seven SARS-CoV-2 waves and one influenza wave (CW 47/2022–01/2023). Standardized mortality ratios (SMR) were calculated by dividing the observed number of deaths (*O*) by the expected number of deaths (*E*) for the years 2020–2023 and for the above mentioned waves. The standard error of SMR was calculated as $\sqrt{O}$ / *E* which is equivalent to the formula by Vandenbroucke for large values of *O* [19]. To calculate the expected number of deaths for each pandemic year, two methods were applied.

In the first method, the average mortality rates from the pre-pandemic years 2016–2019 were first determined for each calendar week (CW) and each age group (0–29, five-year groups (30–34, 35–39 … up to 95 + years). The expected number of deaths for each age group was calculated by multiplying the average mortality rates by the age-specific population figures for each pandemic year. Adding the expected deaths for each age group gave the total number of expected deaths. In the second method, the expected weekly number of deaths was calculated based on extrapolated mortality rates instead of the mean rates observed between 2016 and 2019 in order to reflect increasing life expectancy. Age-specific weekly mortality rates for 2020 were extrapolated using an exponential model *d*_*x*_*(t)* = exp (*a*_*x*_ + *b*_*x*_ (*t* – 2015)), with d_x_ indicating the mortality rate of age group x and t denoting the calendar year. For both methods, SMRs are estimated for all calendar weeks of the years 2020–2023.

In addition to SMR, we calculated the difference between the observed and expected cases for each pandemic year, for each SARS-CoV-2 wave and for the influenza wave at the end of 2022. For better comparison, excess deaths per week were calculated for the pandemic years and the various waves.

Additionally, for five age categories (0–29, 30–59, 60–69, 70–79, and 80 + years) SMRs were calculated separately for all calendar weeks of the years 2020–2023, for the whole years 2020–2023, and for the influenza wave at the end of 2022 using method 1.

Calculations were done using SAS Version 9.4 (SAS Institute, Cary, USA).

## Results

**Fig 1:**
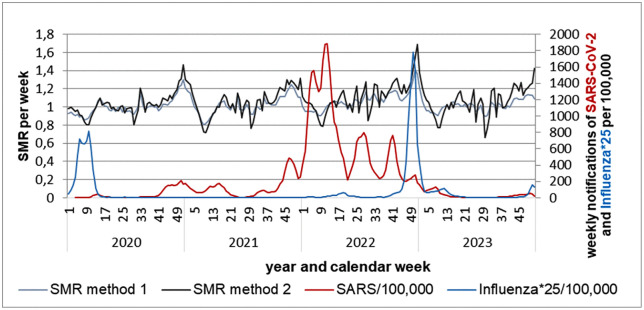
Standardized Mortality Ratios (SMRs) per week 2020 to 2023 by method 1 and method 2 – compared to weekly notification rates of SARS-CoV-2 and influenza per 100,000 of the German population.

Using the constant-mortality rate model (method 1), the largest excess mortality was observed in 2022 (SMR = 1.069 (95% confidence interval (CI): 1.066–1.071; 68,218 excess deaths), whereas no excess mortality was observed in 2020 (Table 1, Fig 1). When the falling trend in mortality in the pre-pandemic years was extrapolated (method 2), there was stronger excess mortality in all years from 2020 to 2023 (Table 1, Fig 1). However, the order of magnitude remained the same. In 2022, using method 2 the SMR was 1.094 (95% CI: 1.092–1.096), with 91,740 excess deaths.

**Table 1:** Standardized mortality ratios in Germany – from 2020 to 2023 and during the individual SARS-CoV-2 waves and the influenza wave in 2022.

		SMR according to Method 1#					SMR according to Method 2##				
		Number of observed deaths	Number of expected deaths	Excess mortality SMR (95% CI)	Excess mortality Difference	Mean excess deaths per week	Number of observed deaths	Number of expected deaths	Excess mortality SMR (95% CI)	Excess mortality Difference	Mean excess deaths per week
Years	2020	975907	978775	0.997 (0.995–0.999)	−2867	−55	975907	963825	1.013 (1.011–1.015)	12082	232
	2021	1019045	995265	1.024 (1.022–1.026)	23780	457	1019045	975474	1.045 (1.043–1.047)	43571	838
	2022	1064232	996014	1.069 (1.066–1.071)	68218	1312	1064232	972492	1.094 (1.092–1.096)	91740	1764
	*2022 CW 1–46*	*914433*	*877007*	*1.043* *(1.041-1.045)*	*37426*	*814*	*914433*	*863423*	*1.059* *(1.057-1.061)*	*51010*	*1109*
	*2022 CW 47–52*	*149799*	*119007*	*1.259* *(1.252-1.265)*	*30792*	*5132*	*149799*	*109069*	*1.373* *(1.366-1.380)*	*40730*	*6788*
	2023	1024408	1006766	1.018 (1.016–1.019)	17642,0	339	1024408	980244	1.045 (1.043–1.047)	44164	849
											
Waves * SARS-CoV-2	1: CW 10–20/2020	210470	212869	0.989 (0.985-0.993)	−2398	−218	210470	214226	0.982 (0.978-0.987)	−3756	−341
	2: CW 40/2020–8/2021	445591	416177	1.071 (1.068-1.074)	29414	2262	445591	396951	1.123 (1.119-1.126)	48640	2316
	3: CW 9–23/2021	277745	292676	0.949 (0.945-0.953)	−14931	−995	277745	297763	0.933 (0.929-0.936)	−20018	−1335
	4: CW 31–51/2021	417921	386955	1.080 (1.077-1.083)	30966	1475	417921	371831	1.124 (1.121-1.127)	46090	2195
	5: CW 52/2021-21/2022	445738	449469	0.992 (0.989-0.995)	−3731	−170	445738	443417	1.005 (1.002-1.008)	2321	105
	6: CW 22–33/2022	230376	212968	1.082 (1.077-1.086)	17408	1451	230376	217747	1.058 (1.054-1.062)	12628	1052
	7: CW 34–46/2022	260493	234567	1.111 (1.106-1.115)	25926	1994	260493	219157	1.189 (1.184-1.193)	41336	3180
Wave Influenza	CW 47/2022–1/2023	175263	139956	1.252 (1.246-1.258)	35307	5043	175263	127581	1.374 (1.367-1.380)	47682	6812

SMR: standardized mortality ratio; CI: confidence interval; CW: calendar week; *waves according to Tolksdorf et. al, and according to own definitions from the end of wave 6 and of wave 7

Wave 1 and 2: SARS-CoV-2 wild type dominant, Wave 3: Alpha type dominant; Wave 4: Delta type dominant; waves 5–7: Omicron type dominant

#method 1: average mortality in the pre-pandemic years 2016–2019 was used for calculating the expected number of deaths; #method 2: an exponential extrapolation from the pre-pandemic years 2016–2019 onwards was used for calculating the expected number of deaths (see method section).

For both methods of estimating the number of expected deaths during the pandemic years, excess mortality was observed in SARS-CoV-2 waves 2, 4, 6 and 7 (and in wave 5 only for method 2) (Table 1). During the influenza wave from calendar week 47 / 2022 to calendar week 1 / 2023, the SMR was higher than that of any SARS-CoV-2 wave as determined by both methods: SMR = 1.252 (95% CI: 1.246–1.258) by method 1, and SMR = 1.374 (95% CI: 1.367–1.380) by method 2. Using method 1, the largest number of excess deaths occurred during the influenza wave (35,307 excess deaths). Using method 2, the number of excess deaths was largest during the SARS-CoV-2 waves 2 and 4 and during the influenza wave (46,090–48,640 excess deaths). The number of excess deaths per week was highest during the influenza wave by both methods. Fig 1 shows that the increase in SMR during the final weeks of 2022 occurred at the same time as a sharp rise in influenza notifications. When an alternative time interval was used for the influenza wave (CW 48 / 2022–2 / 2023), results for excess mortality hardly differed by both methods (e.g., SMR = 1.383 (95% CI: 1.377–1.390 versus SMR = 1.374 (95% CI: 1.367–1.390) by method 2). The high excess mortality at the end of the year 2022 can also be seen when SMRs are estimated for quarters of a year or for 4-week intervals (cf. S4 File).

For each of the five age categories, the SMR during the influenza wave (CW 47 /2022 to CW 1 / 2023) was much higher than the SMR for the whole of 2022: 1.133 (0–29 years), 1.105 (30–59 years), 1.189 (60–69 years), 1.291 (70–79 years) and 1.273 (80 + years), compared to 1.069 for the whole year 2022 (see Table 2). Thus, the highest relative excess mortality during the influenza wave was observed in individuals aged 70 years and over (Fig 2).

**Table 2:** Standardized Mortality Ratios (SMRs) in Germany 2020-2023 by method 1# – in the whole population by age groups.

Year	All		0-29 y		30-59 y		60-69 y		70-79 y		80 + y	
	SMR 95%CI	Difference (p. week)	SMR 95%CI	Difference (p. week)	SMR 95%CI	Difference (p. week)	SMR 95%CI	Difference (p. week)	SMR 95%CI	Difference (p. week)	SMR 95%CI	Difference (p. week)
2020	0.997 (0.995-0.999)	−2867.7 (−55.1)	0.923 (0.901-0.944)	−595.7 (−11.5)	0.970 (0.964-0.977)	−2411.5 (−46.4)	0.998 (0.993-1.004)	−191.9 (−3.7)	1.012 (1.008-1.017)	2445.8 (47.0)	0.996 (0-994-0.999)	−2114.4 (−40)
2021	1.024 (1.022-1.026)	23780.3 (457.3)	0.945 (0.923-0.966)	−426.3 (−8.2)	1.017 (1.010-1.024)	1388.7 (26.7)	1.053 (1.048-1.059)	6400.4 (123.1)	1.066 (1.061-1.070)	12559.2 (241.5)	1.006 (1.004-1.009)	3858.2 (74.2)
2022	1.069 (1.066-1.071)	68217.5 (1311.9)	0.999 (0.976-1.021)	−10.2 (−0.2)	0.999 (0.992-1.006)	−62.2 (−1.2)	1.053 (1.048-1.059)	6523.7 (125.5)	1.098 (1.093-1.102)	18363.1 (353.1)	1.072 (1.070-1.075)	43403.0 (834.7)
2023	1.018 (1.016-1.019)	17642.1 (339.3)	0.955 (0.933-0.977)	−347.2 (−6.7)	0.959 (0.953-0.966)	−3182.9 (−61.2)	1.012 (1.007-1.018)	1529.4 (29.4)	1.059 (1.054-1.064)	11190.5 (215.2)	1.014 (1.011-1.017)	8452.3 (162.5)
2022 Influenza wave *	1.252 (1.246-1.258)	35307.3 (5043.0)	1.133 (1.067-1.199)	133.1 (19.0)	1.105 (0.085-1.125)	1117.5 (159.6)	1.189 (1.172-1.205)	3190.0 (455.7)	1.291 (1.277-1.305)	7546.3 (1078.0)	1.273 (1.265-1.280)	23320.4 (3331.5)

*CW 37/2022 – CW 1/2023

#method 1: average mortality in the pre-pandemic years 2016–2019 was used for calculating the expected number of deaths

**Fig 2:**
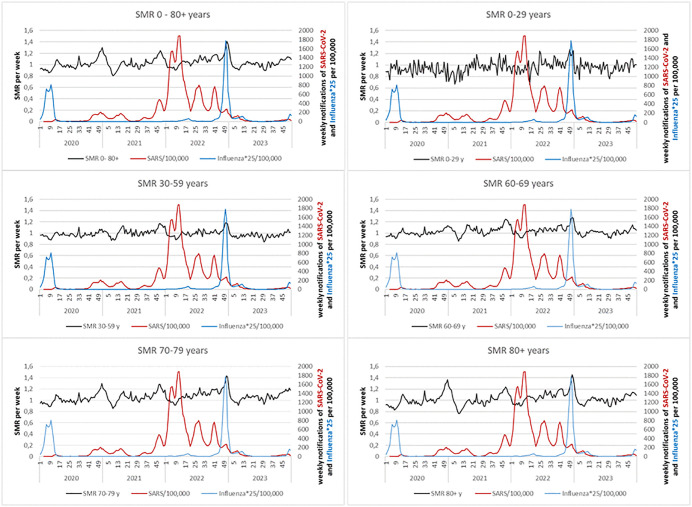
Standardized Mortality Ratios (SMRs) per week 2020 to 2023 by method 1# in the German population and in various age groups – compared to weekly notification rates of SARS-CoV-2 and Influenza per 100,000 population.

## Discussion

Internationally, very different levels of excess mortality have been reported in various countries during the pandemic. Possible causes discussed include differences in the age structure of the population [6], differences in the economic structures of the countries [3], and differences in the intensity of non-pharmacological measures and the behavior of the population [20]. However, as the focus of our work is on Germany and the question of high excess mortality in 2022, these issues of international comparison are not addressed further.

Our results for whole pandemic years are in line with earlier findings from Germany and from Frankfurt am Main [10–12,21]: in Germany, excess mortality was strongest in 2022, higher than in the other three pandemic years. As expected, excess mortality was generally higher when increase in life expectancy was taken into account.

Our hypothesis derived from the former small scale study in Frankfurt am Main was confirmed for Germany: excess mortality at the end of 2022 coincided with an influenza wave. This result was robust: it was obtained using various statistical methods and was found not only in the total population but also in the five age groups examined separately. The relative excess mortality during the influenza wave in the final weeks of 2022 was highest for persons older than 70 years.

### Hypotheses for Excess Mortality in Germany in 2022

Rockenfeller and Günter had hypothesized that the high excess mortality in 2022, especially in different age groups, could be due to early experiences of deprivation in life (war and/or post-war era) and possibly to immune dysregulation caused by the repeated vaccinations against SARS-CoV-2, and called for further hypothesis-driven investigations [21]. Kuhbandner and Reitzner concluded from the high excess mortality in 2022 that there was an additional excess mortality driver and, given the high correlations between excess mortality in 2022 and the vaccination rate against SARS-CoV-2, assumed that the high excess mortality in 2022 might be an unintended effect of vaccination. However, they also did not rule out other previously neglected mortality drivers [11].

Our investigation showed that the high SMR of 1.069 (method one) for the year 2022 as a whole was (partly) caused by the increase in the SMR to 1.252 in calendar weeks 47/22–1/2023 – parallel to a significant increase in influenza reports. In the previous calendar weeks 1–46/2022, the SMR was 1.043 (95% CI 1.041–1.045). Regardless of the statistical method chosen, almost half of the excess deaths in Germany in 2022 occurred in the last weeks of 2022. This (at least partially) confirms our hypothesis of a connection between the high SMR in 2022 and the influenza wave at the end of 2022.

The Europe wide data from EURO MOMO also showed the same pattern, albeit using a different statistical method: the highest excess mortality rates in 2022 occurred in calendar weeks 48/2022–2/2023 [18]. This coincided with high influenza activity in Europe, which reached near-pandemic levels, but started and peaked earlier than in former years (mostly influenza A(H3N2) [22].

The question arose as to whether this high excess mortality was caused by particularly strong virus circulation or by a particularly virulent virus strain.

### Methodical Aspects of Notification data for SARS-CoV-2 and Influenza

The much lower reporting figures for influenza compared to SARS-CoV-2 reports cannot be directly interpreted as an indication of a much lower circulation of influenza viruses compared to SARS-CoV-2. Depending on the availability of tests and the mandatory testing requirements in place, very high 7-day incidence rates for SARS-CoV-2 were achieved throughout the pandemic. At the end of 2022 testing for SARS-CoV-2 was still mandatory in Germany, so that not only those who were actually ill but also those who tested positive for SARS-CoV-2 without showing any symptoms were recorded. This limits comparability with reports on other infectious diseases such as influenza. However, irrespective of the number of reports for SARS-CoV-2 and influenza, these can be used effectively to define waves.

Moreover, since most cases of influenza are usually diagnosed based on clinical symptoms alone, while only laboratory-confirmed cases are subject to mandatory reporting, a significant underreporting of influenza in the reporting system must be assumed [23]. Furthermore, comparisons of influenza notifications with previous years are limited by different surveillance activities/studies and by reference definitions that were changed on January 1, 2019 [23]. By including all laboratory-confirmed cases in the new reference definition – even in cases where the clinical picture was not fulfilled or unknown – an 26% increase in notification cases was obtained in 2019 and 2020, whereas from 2021 onwards the increase was about a third (30–34%) [23–25].

### Surveillance Data for Respiratory Diseases – SARS-CoV-2 and Influenza

Therefore, the Robert Koch-Institute (RKI) emphasizes that the data collected as part of the AGI’s (Arbeitsgemeinschaft Influenza, i.e., working group influenza) sentinel surveillance [26] are more suitable for assessing the burden of respiratory disease than the notified laboratory data [27]. These surveys were established after the last pandemic in 2009 and recommended in the National Pandemic Plan [28,29] as the basis for pandemic management. In addition, they provide the advantage of data collected over many years using an unchanged method, which allows for a “historical comparison” with the pre-pandemic period [30]. According to these surveillance data significantly fewer respiratory diseases and influenza-like illnesses were reported during the pandemic than in the pre-pandemic years, and doctor consultations and hospitalizations for respiratory diseases remained below or in line with previous years. At the end of 2022, reported respiratory diseases and influenza-like illnesses rose to higher levels than in all pre-pandemic years with data available, whereas doctor consultations and hospital admissions for respiratory diseases were comparable to previous winter seasons. This was observed even though various non-pharmacological measures to prevent infections were still in place at the time, albeit to varying degrees in the different federal states of Germany, such as mandatory testing in schools or the requirement to wear FFP2 masks on public transport. Concomitant virological surveillance showed that this was mainly due to influenza viruses. The positive rate for influenza viruses was 40%, for parainfluenza virus it was about 20%, while that for SARS-CoV-2, rhinoviruses, and respiratory syncytial viruses was less than 10% in respective cases [31].

In line with these surveillance data and our mortality data, the data of the infection radar published by the Federal Ministry of Health, starting in 2020, show by far the highest number of doctor visits and hospitalizations due to respiratory diseases as well as a higher intensive care bed utilization in weeks 50−52 /2022 during the influenza wave than during the entire SARS-CoV-2 pandemic years and waves – until today [32].

### Excess Mortality Data – SARS-CoV-2 and Influenza

Excess mortality is influenced by many factors: in addition to the extent of virus circulation, the virulence of the pathogen, and the immunity of the population, whether through vaccination or previous infections, as well as the protective measures taken – while other influencing factors cannot be ruled out. Hence, wave-level SMR comparisons do not equate to comparisons of intrinsic virulence or “severity,” given differences in immunity, age/risk composition of infections, treatment availability, testing/ascertainment, and concurrent respiratory pathogen circulation. Thus, the higher SMR during the influenza wave in late 2022 compared to the lower SMR values during the SARS-CoV-2 waves 5−7 does not necessarily indicate a higher pathogenicity of the circulating influenza viruses compared to the circulating SARS-CoV-2 (Omicron variant).

In the first phase of the pandemic (2020-January 2021), the 30-day mortality rate among patients hospitalized with COVID-19 was 2.9 times [33], and 2.3 times [34] higher than among patients hospitalized with influenza. At that time, the wild-type SARS-CoV-2 was circulating and affecting a population that was immunologically naïve to SARS-CoV-2. In 2022, the much less virulent but significantly more contagious Omicron variant of SARS-CoV-2 was circulating, and the majority of the population displayed increasingly high vaccine- and infection-derived protection. Against this background, a recent study in Switzerland compared the 30-day mortality of 2,901 patients admitted with healthcare-associated COVID-19 between February 1, 2022, and April 30, 2023, with 868 patients admitted with healthcare-associated influenza between November 1, 2018, and April 30, 2023. The COVID patients were infected with the Omicron variant, and 84%of them had been vaccinated against SARS-CoV-2, 81% of whom had received at least 3 doses and 10% more than 3 doses. Under these conditions, the 30-day mortality rate among hospitalized COVID-19 patients was 6.2%. This was no higher than among hospitalized influenza patients with a 30-day mortality rate of 6.1% [35].

Throughout Europe, excess mortality in the winter of 2022/23 was elevated. This occurred at a time when COVID‑19 measures had already ended in many countries (but not in Germany) and influenza activity was high in many countries [22]. The excess mortality was similar to that seen in the winter periods of 2018/19, 2017/18, 2016/17, and 2014/15 [18].

### Strengths and limitations

Our study has several strengths. We used mortality data the survey of which was already completed by the Federal Statistical Office. Our analyses were done on a week-specific basis which allowed the distinct analyses of single SARS-CoV-2 and influenza waves. Furthermore, the estimation of expected numbers of deaths using average mortality was supplemented by an analysis which took increasing life expectancy into account. An important strength of our study was that the association between the influenza wave and excess mortality in 2022 was robust regardless how expected deaths were calculated.

A limitation of our study is that the estimation of SMR strongly depends on methodical choices so that our analyses are based on arbitrary decisions on the choice of age categories, the selection of pre-pandemic years, the method of extrapolation of mortality from pre-pandemic to pandemic years etc. However, the focus of our study was not estimation of SMRs during the SARS-CoV-2 pandemic. Rather, we confirmed our hypothesis that the influenza wave at the end of 2022 was associated with strong excess mortality. Our analyses suggest that this result does not depend on specific methodical choices. A further limitation is that we used only 52 calendar weeks because the tables offered by the Federal Statistical Office do not include CW 53 for death cases on a weekly basis for the years 2016–2023 except for the year 2020. Using only 52 calendar weeks, we also could not take leap years (2016, 2020) into account. However, it is very unlikely that the lack of CW53 has a strong impact on the results.

## Conclusion

Our study confirmed our hypothesis: the high excess mortality in 2022 compared to other pandemic years was mainly due to the marked excess mortality in the last weeks of the year. This was associated with an influenza wave at the end of 2022. The result was robust: it was obtained using various statistical methods and not only in total population but also in the five age groups examined. This finding, which had been overseen due to the previous focus on COVID-19 and had already been described in a small-scale analysis in Frankfurt am Main, was thus confirmed for the first time for the whole of Germany.

During the pandemic, in Germany measures were essentially based on SARS-CoV-2 notification data. However, the high disease burden and mortality in the last weeks of 2022 was not reflected in the SARS-CoV-2 reporting data, but it was evident early on in the German surveys established after the last pandemic in 2009 and in the European mortality registry EURO MOMO, especially when compared with the “historical data” collected before the pandemic. These findings support the systematic integration of surveillance systems recommended for preparedness planning with notification-based indicators in future respiratory epidemic responses.

## Supporting information

S1 File Population_Germany_2016_2023. Population of Germany by year and age group.
(XLSX)

S2 File Death_cases_Germany_2016_2023. Death cases in Germany by year, calendar week and age group.
(XLSX)

S3 File Notifications_SARS-CoV-2 & Influenza. Notifications of SARS-CoV-2 and influenza cases per 100,000 by year and calendar week for 2020–2023 for Germany.
(XLSX)

S4 File Standardized mortality ratios by months and quarters of a year. Observed and expected death cases, and standardized mortality ratios (SMRs) (95% confidence intervals) by quarters of a year and by month (2020–2023) for Germany.
(DOCX)
