# Supplementary material for: All‑cause excess mortality in Germany peaked during the late‑2022 influenza period, exceeding peaks during SARS‑CoV‑2 waves (2020–2023)
Source: PLoS One. 2026 Jun 17;21(6):e0335982. doi: 10.1371/journal.pone.0335982 (PMC13274847; doi:10.1371/journal.pone.0335982)
Supplement: S4 File — Observed and expected death cases, and standardized mortality ratios (SMRs) (95% confidence intervals) by quarters of a year and by month (2020–2023) for Germany. (DOCX) [file pone.0335982.s004.docx]

**Standardized Mortality Ratios (SMR, 95% CI) by quarters of the years 2020 to 2023** ^a^

| Year | Quarter | Deaths  observed | Deaths expected | SMR (observed/  expected) | SMR  95%CI (lower CL) | SMR 95%CI (upper CL) | Difference (observed-expected) |
| --- | --- | --- | --- | --- | --- | --- | --- |
| 2020 | I | 251905 | 275998 | 0.913 | 0.909 | 0.916 | -24093 |
|  | II | 233050 | 232407 | 1.003 | 0.999 | 1.007 | 643 |
|  | III | 223806 | 226103 | 0.990 | 0.986 | 0.994 | -2297 |
|  | IV | 267146 | 244266 | 1.094 | 1.090 | 1.098 | 22880 |
| 2021 | I | 270837 | 280713 | 0.965 | 0.961 | 0.968 | -9876 |
|  | II | 238633 | 236246 | 1.010 | 1.006 | 1.014 | 2387 |
|  | III | 229276 | 229880 | 0.997 | 0.993 | 1.001 | -604 |
|  | IV | 280299 | 248426 | 1.128 | 1.124 | 1.132 | 31873 |
| 2022 | I | 269131 | 280972 | 0.958 | 0.954 | 0.961 | -11841 |
|  | II | 246615 | 236398 | 1.043 | 1.039 | 1.047 | 10217 |
|  | III | 252056 | 230052 | 1.096 | 1.091 | 1.100 | 22004 |
|  | IV | 296430 | 248593 | 1.192 | 1.188 | 1.197 | 47837 |
| 2023 | I | 280492 | 284075 | 0.987 | 0.984 | 0.991 | -3583 |
|  | II | 243488 | 238938 | 1.019 | 1.015 | 1.023 | 4550 |
|  | III | 227615 | 232529 | 0.979 | 0.975 | 0.983 | -4914 |
|  | IV | 272813 | 251224 | 1.086 | 1.082 | 1.090 | 21589 |

^a^ SMRs have been estimated according to method 1.

**Standardized Mortality Ratios (SMRs, 95% CI) for 4-week intervals of the years 2020 to 2023** ^a^

| Year | 4-week interval | Deaths  observed | Deaths expected | SMR (observed/  expected) | SMR  95%CI (lower CL) | SMR 95%CI (upper CL) | Difference (observed-expected) |
| --- | --- | --- | --- | --- | --- | --- | --- |
| 2020 | 1 | 76071 | 82431 | 0.923 | 0.916 | 0.929 | -6360 |
|  | 2 | 77413 | 86586 | 0.894 | 0.888 | 0.900 | -9173 |
|  | 3 | 98421 | 106982 | 0.920 | 0.914 | 0.926 | -8561 |
|  | 4 | 78982 | 74749 | 1.057 | 1.049 | 1.064 | 4233 |
|  | 5 | 69735 | 71212 | 0.979 | 0.972 | 0.987 | -1477 |
|  | 6 | 84333 | 86446 | 0.976 | 0.969 | 0.982 | -2113 |
|  | 7 | 66023 | 70182 | 0.941 | 0.934 | 0.948 | -4159 |
|  | 8 | 72182 | 70068 | 1.030 | 1.023 | 1.038 | 2114 |
|  | 9 | 85601 | 85853 | 0.997 | 0.990 | 1.004 | -252 |
|  | 10 | 71457 | 71961 | 0.993 | 0.986 | 1.000 | -504 |
|  | 12 | 77500 | 74327 | 1.043 | 1.035 | 1.050 | 3173 |
|  | 13 | 118189 | 97977 | 1.206 | 1.199 | 1.213 | 20212 |
| 2021 | 1 | 96254 | 83839 | 1.148 | 1.141 | 1.155 | 12415 |
|  | 2 | 82191 | 88072 | 0.933 | 0.927 | 0.940 | -5881 |
|  | 3 | 92392 | 108802 | 0.849 | 0.844 | 0.855 | -16410 |
|  | 4 | 76808 | 75994 | 1.011 | 1.004 | 1.018 | 814 |
|  | 5 | 72912 | 72397 | 1.007 | 1.000 | 1.014 | 515 |
|  | 6 | 88913 | 87854 | 1.012 | 1.005 | 1.019 | 1059 |
|  | 7 | 69480 | 71354 | 0.974 | 0.966 | 0.981 | -1874 |
|  | 8 | 69233 | 71240 | 0.972 | 0.965 | 0.979 | -2007 |
|  | 9 | 90563 | 87286 | 1.038 | 1.031 | 1.044 | 3277 |
|  | 10 | 77306 | 73172 | 1.056 | 1.049 | 1.064 | 4134 |
|  | 12 | 87114 | 75591 | 1.152 | 1.145 | 1.160 | 11523 |
|  | 13 | 115879 | 99663 | 1.163 | 1.156 | 1.169 | 16216 |
| 2022 | 1 | 80860 | 83921 | 0.964 | 0.957 | 0.970 | -3061 |
|  | 2 | 82733 | 88156 | 0.938 | 0.932 | 0.945 | -5423 |
|  | 3 | 105538 | 108894 | 0.969 | 0.963 | 0.975 | -3356 |
|  | 4 | 80146 | 76054 | 1.054 | 1.047 | 1.061 | 4092 |
|  | 5 | 74287 | 72446 | 1.025 | 1.018 | 1.033 | 1841 |
|  | 6 | 92182 | 87898 | 1.049 | 1.042 | 1.056 | 4284 |
|  | 7 | 78458 | 71410 | 1.099 | 1.091 | 1.106 | 7048 |
|  | 8 | 78728 | 71295 | 1.104 | 1.097 | 1.112 | 7433 |
|  | 9 | 94870 | 87346 | 1.086 | 1.079 | 1.093 | 7524 |
|  | 10 | 85685 | 73220 | 1.170 | 1.162 | 1.178 | 12465 |
|  | 12 | 82397 | 75639 | 1.089 | 1.082 | 1.097 | 6758 |
|  | 13 | 128348 | 99733 | 1.287 | 1.280 | 1.294 | 28615 |
| 2023 | 1 | 90087 | 84854 | 1.062 | 1.055 | 1.069 | 5233 |
|  | 2 | 83527 | 89132 | 0.937 | 0.931 | 0.943 | -5605 |
|  | 3 | 106878 | 110089 | 0.971 | 0.965 | 0.977 | -3211 |
|  | 4 | 79302 | 76887 | 1.031 | 1.024 | 1.039 | 2415 |
|  | 5 | 74752 | 73223 | 1.021 | 1.014 | 1.028 | 1529 |
|  | 6 | 89434 | 88829 | 1.007 | 1.000 | 1.013 | 605 |
|  | 7 | 68681 | 72181 | 0.952 | 0.944 | 0.959 | -3500 |
|  | 8 | 71021 | 72068 | 0.985 | 0.978 | 0.993 | -1047 |
|  | 9 | 87913 | 88280 | 0.996 | 0.989 | 1.002 | -367 |
|  | 10 | 76802 | 73997 | 1.038 | 1.031 | 1.045 | 2805 |
|  | 12 | 82949 | 76439 | 1.085 | 1.078 | 1.093 | 6510 |
|  | 13 | 113062 | 100789 | 1.122 | 1.115 | 1.128 | 12273 |

^a^ SMRs have been estimated according to method 1.
